# Effects of Maternal Care During Rearing in White Leghorn and Brown Nick Layer Hens on Cognition, Sociality and Fear

**DOI:** 10.3390/ani9070454

**Published:** 2019-07-18

**Authors:** Susie E. Hewlett, Rebecca E. Nordquist

**Affiliations:** 1 Department of Biological Sciences, Macquarie University, North Ryde, NSW 2109, Australia; 2 Behavior and Welfare in Farm Animals Research Group, Department of Farm Animal Health, Faculty of Veterinary Medicine, Utrecht University, 3584CL Utrecht, The Netherlands

**Keywords:** Maternal care, gallus gallus, hen, spatial memory, fear, behavior, sociality, chicken

## Abstract

**Simple Summary:**

Chickens raised to lay eggs are housed from hatch in groups of animals of the same age, and without maternal care from a broody hen. There are several hybrid lines of hens used in egg farming, each of which show their own behavioral profile. Both the presence (or absence) of a mother hen and genetics may affect cognition, social interactions and fear. In this study, we showed that in our tests, genetics have a strong effect on fear and sociality. Maternal care had very little effect on any of the tests used. The strong effect of genetic background highlights that changes made to increase welfare need to consider the genetics of the chicken in question. The lack of effect of maternal care may indicate that breeds of chickens used in current farming practices were inadvertently selected to respond very little to maternal care.

**Abstract:**

Both genetic background and maternal care can have a strong influence on cognitive and emotional development. To investigate these effects and their possible interaction, White Leghorn (LH) and Brown Nick (BN) chicks, two hybrid lines of layer hen commonly used commercially, were housed either with or without a mother hen in their first five weeks of life. From three weeks of age, the chicks were tested in a series of experiments to deduce the effects of breed and maternal care on their fear response, foraging and social motivation, and cognitive abilities. The LH were found to explore more and showed more attempts to reinstate social contact than BN. The BN were less active in all tests and less motivated than LH by social contact or by foraging opportunity. No hybrid differences were found in cognitive performance in the holeboard task. In general, the presence of a mother hen had unexpectedly little effect on behavior in both LH and BN chicks. It is hypothesized that hens from commercially used genetic backgrounds may have been inadvertently selected to be less responsive to maternal care than ancestral or non-commercial breeds. The consistent and strong behavioral differences between genetic strains highlights the importance of breed-specific welfare management processes.

## 1. Introduction

In current commercial production systems for layer hens, chicks of one of several common layer hybrids are hatched and raised in homogenous groups in terms of age, and never experience maternal care [1]. As seen in mammals, precocial bird species such as chickens are known to express high levels of maternal care in the first weeks of their offsprings’ lives, which has been most extensively studied in quail (see [2,3], but see Vallortigara et al. [4] for chicken). During this period, the mother provides lessons in successful foraging and social interactions, as well as warmth and safety while communicating with the chicks through calling and allowing chicks to huddle under her while brooding. In mammals, all these components of maternal care greatly impact the emotional stability, cognition and sociability of the offspring [5,6,7]. As adults, chickens usually go on to live in structured social aggregations [8]. However, throughout commercially housed chicken populations, abnormal and aggressive feather pecking behavior is prevalent. Understanding the effects of maternal care on chick development in terms of fear, sociability, and cognition may reveal another method for tackling feather pecking and improve the welfare of animals on farm later in life. Perré et al. [9] and Shimmura et al. [10] both found that brooded pullets and chicks, respectively, were more active and exploratory compared to non-brooded conspecifics. However, while Shimmura and colleagues found a difference in the fear responses of brooded and non-brooded chicks, Perré and colleagues found no differences between brooded and non-brooded pullets [9]. Interestingly, Perré and colleagues noted a clear linear hierarchy among brooded pullets, coinciding with reduced feather pecking and a propensity to seek and maintain contact with familiar conspecifics. Overall, however, interactions between brooded pullets were more antagonistic than among non-brooded pullets. Various chicken breeds and hybrids also show different behavioral fingerprints such as high or low fear [11], social and cognitive preferences and abilities, respectively [12,13,14]. There may also be an interaction between genetic background and the behavioral effects of maternal care, as Versace et al. [15] report variation in the response to social stimuli of chicks from different breeds.

There is currently a limited number of studies on maternal care effects on galliform cognition and behavior but of the studies done, the main focus is on fear and stress behavior. An early study found that presenting a maternal odorant lowered broiler chick fear behavior when faced with isolation and novelty [16], and more recently, the use of dark brooders to mimic aspects of maternal care also leads to less fear expression later in life [17]. In laying hens, the genetic background of the chicken also plays a strong role in the development of a strong fear response, as studies examining the effects of breed on behavioral and physiological fear responses often find Leghorns to be more fearful compared with other domestic breeds, (see [18]). Fraisse and Cockrem [19] examined physiological and behavioral responses to stress and found the Leghorns had a greater plasma corticosterone response to handling than Brown layer Hyline hens. However, when comparing Leghorns to their ancestral kin, both Schutz et al. [20] and Campler et al. [21] found Leghorns to be less afraid than jungle fowl in novel object testing. Rodenburg et al. [22] found brooded chicks from a selection line derived from White Leghorn to be more exploratory and less afraid compared to chicks from the same line not raised by a mother hen, indicating that both genetics and maternal care can impact chick fear behavior. Interestingly, Angevaare et al. [23] saw little effect of maternal care on fear or other behavioral measures in Silver Nick layers, which may indicate the variable long-term effects of maternal care depending on genetics. When raised by mothers that were genetically selected for greater fearfulness, quail chicks in turn showed more fearfulness. This effect was more prominent in chicks from the strain selected for higher fearfulness than in the strain selected for lower fearfulness [24], further highlighting the importance of both genetics and maternal care, and the potential for the two factors to interact.

The positive effect of maternal care on social behaviors in offspring has been extensively studied in mammals (Reviewed in: [6,25,26,27,28,29,30]) and includes increased aggression following deprivation of maternal contact when young [31], and less defensive behavior towards an unfamiliar conspecific when raised by a mother providing high levels of maternal care [32]. Appropriate social behavior is highly relevant for group-housed animals on a farm, but the effects of early life experience with maternal care on laying hen social behavior in adulthood is understudied. The use of dark brooders, mimicking being under a mother’s wings, has been demonstrated to reduce feather pecking behavior in adult layer hens [33], and studies in quail have found permanent changes in chick social motivation due to the behavior of the mother quail [34]. Moreover, alterations in the neurochemistry of social brain circuitry has been linked to maternal care in chickens [35,36], and may lead to permanent behavioral alterations in the chicks. Given the overwhelming evidence for the effects of maternal care on social behavior in mammals and the importance of the social environment for chickens to thrive on farm, more research is warranted in chickens on this topic.

Sociability and the expression of social behaviors are also partially dependent upon the genetic makeup of the individual, as has been demonstrated in chickens in a handful of studies. When comparing four lines (high productivity vs. low productivity lines and brown-egg and white-egg variants in each), the high productivity lines showed lower social motivation and the white-egg lines showed more social motivation than brown-egg lines [13]. The white-egg, high-productive Hyline line has also been shown to engage in fewer social behaviors than the ancestral kin Jungle Fowl, and the Swedish Bantam (a backyard chicken unselected for production traits; [37]).

The level and quality of a mother’s care is also known to have a profound effect on the cognitive abilities of her offspring in mammals. When female mice provided with two mothers to increase maternal care experience, these offspring performed better in an object discrimination task over the long-term compared to pups raised with a single mother [29]. Male rats separated from their mother showed impaired spatial, non-spatial, reference, and working memory compared to those only briefly separated during ontogeny [28]. In support, Hulshof et al. [38] found that maternally deprived rats had fewer new-born cells in the dentate gyrus of the hippocampus, an area strongly linked to spatial and working memory. In congruence, the vocalizations of a mother hen positively affect learning and memory in young chicks [39] and rearing with a foster hen has also been found to alter hippocampal lateralization [40], potentially modifying learning and memory capabilities.

Variations in chicken cognitive abilities have also been observed across genetic backgrounds. Tommasi and Vallortigara [41] showed that White Leghorn chicks could be conditioned to a specific place for a food reward and that the birds used various spatial cues to locate the reward. In a different study, layer hen chicks from a Rhode Island and Brown layer cross were found to successfully locate food rewards after training with magnetic cues, while a more genetically mixed (including White Leghorn) meat breed could not locate the food after training [42]. A follow-up study compared Lohmann Brown chicks with pure White Leghorns and also found the brown layers successfully learnt the magnetic cues to retrieve a reward whereas the white layers were unsuccessful [43]. Genetic differences in learning have also been demonstrated in layer hens using Skinner boxes, with high-productivity lines showing faster acquisition of an operant learning task than low-productivity lines [12].

In the present study, the effects of maternal care on female chicks from two hybrid lines (White Leghorn and Brown Nick) commonly used in commercial egg laying farming were tested. The two lines were either raised with or without a foster hen, and the four treatment groups were then tested in a series of behavioral tests to assess their emotional reactivity to novelty, social motivation and cognitive abilities. We predicted that the chicks raised with a mother hen would show less intense fear behavior, more exploratory behavior in novel situations, and better performance in a cognitive task compared to the chicks raised without a hen. We further predicted that the White Leghorns would show more fearfulness, greater sociability, and perform worse in the cognitive task than the Brown Nicks.

## 2. Materials and Methods

All procedures were approved and carried out under the guidelines of the Utrecht University DEC (animal ethics committee) in accordance with the recommendations of the EU directive 2010/63/EU, under protocol 2010.I.06.090.

### 2.1. Animals

Twenty H&N Brown Nick (BN) and 20 Lohmann classic White Leghorn (LH) female chicks were hatched at Verbeek Hatchery, Lunteren, The Netherlands and transported to the Utrecht University farm the same day. Three Silkie Bantam hens, a breed known to reliably express high levels of maternal behavior [10], were housed at the university farm 4 weeks prior to the chicks’ arrival.

During these four weeks, the hens habituated to their new environment and were encouraged to brood using chalk eggs. Chicks arrived and were randomly split across the four adjacent home pens so that 5 chicks of each line were in each pen. The two middle pens also housed a broody Silkie hen acting as a foster mother, creating four treatment groups: Brown Nicks raised by a hen (BN HEN), Brown Nicks raised without a hen (BN NO), White Leghorns raised with a hen (LH HEN), and White Leghorns raised without a hen (LH NO). During the first 24 h, the foster hens were informally monitored for maternal behavior towards the chicks in the form of brooding [4], and absence of any aggression towards them [44,45]. We ran the experiment during the spring and summer and the rearing pens were maintained under natural light conditions. During the first week, the chicks were left to habituate to their home pens, and in the two center pens, to bond with their foster mothers. All chicks were vaccinated against avian diphtheria at the end of week 3.

All chicks were weighed twice a week up to and including week 9, and once a week from then on. All chicks gained weight on each weigh-in except during weeks 5 and 6, when some chicks became ill with coccidiosis necatrix (see below for details) and were treated by a trained veterinarian. The chicks’ weights were always within the healthy weight range for their age according to the management guidelines for their respective hybrid line [46,47].

### 2.2. Housing

The four pens were side by side and visually separated by wire mesh and cardboard. Starter chick food (Besterfood pluimvee voeders, Ede, The Netherlands) and water were provided ad libitum and all pens were kept under natural light conditions during spring and summer. All pens were provided with heat lamps that produced a ground temperature of at least 28 °C directly underneath for the first 2 weeks of the experiment. During later weeks, a heating element at the rear of the pen produced a localized ground temperature of at least 22 °C. In the rest of the pen, the temperature varied between a minimum of 10 to maximum of 25 °C over the course of the experiment, allowing the animals to behaviorally regulate temperature. Mixed grain (Besterfood pluimvee voeders, Ede, The Netherlands) was scattered in each pen daily from week 3 onwards. During weeks 1 and 2, each chick was handled daily by the experimenter to habituate them to the person and procedure. Chicks were also marked with paint (Kruuse, Denmark) for individual recognition during testing. At the end of week 5 the foster mothers were removed from the home pens. At this age, the chicks were informally observed to perch more often than to rest with the foster hen, in accordance with behavior found by Shimmura et al. [10].

### 2.3. Testing

The testing period ran from 3 to 12 weeks of age. All testing was done between 08.00 and 17.00 in a testing room located adjacent to the home pens. The test room measured 3.1 × 2.9 m, had no windows and was illuminated by two heat lamps. A random testing order was made using Microsoft excel 2003 before each test. Data were scored live from a TV monitor in an adjacent room connected to a video camera above the test apparatus. Chicks were returned to their home pen immediately after each test. The observer was usually blind to the rearing treatment (with hen or without) during testing but could visually identify the strain of chick being tested.

#### 2.3.1. Open Field Test

**Figure 1:**
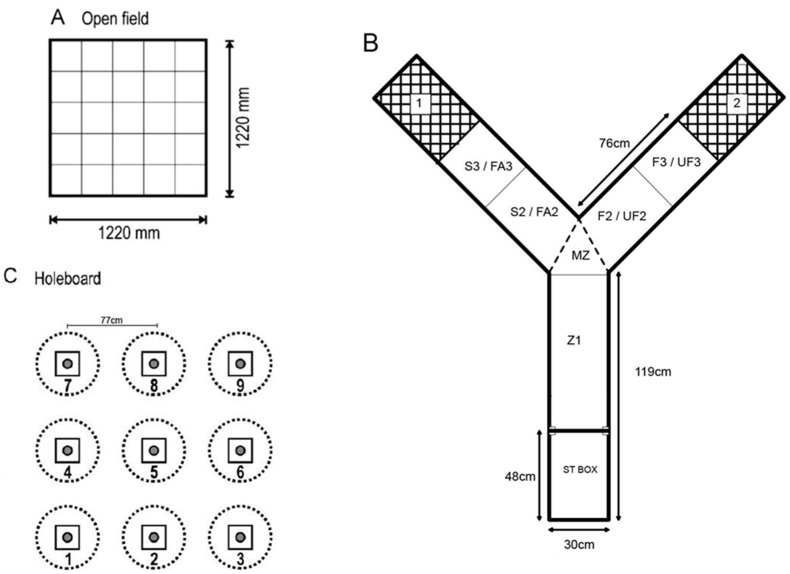
Schematic diagrams of the behavioral apparatus used. (**A**) A birds-eye view of the open field apparatus. All small squares measured 25 cm × 25 cm. A net was draped over the top to prevent escape. (**B**) A birds-eye view of the Y-maze apparatus. For the social versus foraging test, 1 would contain either a pair of pen mates or a foraging zone with scattered feed, and 2 contained the opposite to 1. Each subject chick was randomly assigned 1 = social stimulus or 1 = foraging stimulus. S3 = social zone, S2 = social arm zone, F2 = feeding arm zone, F3 = zone in proximity to feeding zone. For the social recognition test, 1 would contain either a pair of pen mates or a pair of unfamiliar conspecifics from another treatment group, and 2 contained the opposite to 1. Each subject chick was randomly assigned 1 = familiar stimulus or 1 = unfamiliar stimulus. FA3 = familiar zone, FA2 = familiar arm zone, UF2 = unfamiliar arm zone, UF3 = unfamiliar zone. In both tests: MZ = midzone, Z1 = zone 1, ST BOX = start box. (**C**) A birds-eye view of the holeboard apparatus. Circles were drawn with chalk and had a diameter of 50 cm. The distance between each center of the cup (shaded small circles) was 77 cm. The 9 cm × 9 cm squares were medium density fiberboard attached to the cup to keep them steady.

In week 3, chicks were tested once for 10 min in an open field apparatus (for details: [42]) (Figure 1A). The chick was placed in the center of the apparatus while the lights were off, and recording began once the lights were turned on. Latency to walk, number of chalk lines crossed, attempts to fly, and number of distress calls was recorded during the 10-min test. All 40 subjects were tested within 2 days. Apart from latency to walk data, two LH HEN chicks were assigned a missing value due to escaping from the apparatus before the end of the test.

#### 2.3.2. Voluntary Human Approach Test

In addition, in week 3, chicks were tested once for 10 min in a voluntary human approach test. A familiar human sat motionless in the far corner of the testing room holding out a grape. The chick was placed in the opposite corner and another experimenter scored behavior from the TV monitor during the 10-min test. The room was divided into start zone 1 (1.3 × 1.6 m) where the chick was first placed, zone 3 which represented an arc of 80 cm around the human, and zone 2 for the area between zones 1 and 3. Latency to walk and enter each zone, total time in each zone, and time spent on the human was recorded and classed as the chick being unafraid of the human. All 40 subjects were tested within 2 days.

#### 2.3.3. Social Versus Foraging Y-Maze

In weeks 4 and 5, each chick was tested five times over five days for 10 min in the social versus foraging test adapted from Vaisanen and Jensen [48] (Figure 1B). The test animal was placed in the start box and testing began once the door had been lifted via a pulley system. Latency to leave the start box and enter each zone, total time spent in each zone, and the number of lines crossed was recorded in each trial. Each chick was randomly assigned either the left or the right arm as the social stimulus goal box and this remained the same throughout the trials. The other arm contained mixed grain scattered among wood shavings, simulating their pen floor. To test for the development of an arm preference, both arms of the maze were empty during the fifth trial. The familiar social stimulus was a pair of chicks from each pen who were not tested, hence N = 32. One LH HEN was euthanized and 4 chicks in the same pen were not tested after trial 2 due to activity alterations from an anticoccidial treatment (Baycox, Bayer B.V. Kiel, Germany).

#### 2.3.4. Social Recognition Y-Maze

In week 6, each chick was tested four times for 10 min in a social recognition test assessing their ability to distinguish penmates from unfamiliar chicks and their motivation to ‘socialize’. All chicks were tested daily over four days. The Y-maze apparatus used is the same as that used in the test above (Figure 1B) with the exception that the second goal box contained two unfamiliar chicks behind a wire mesh barrier instead of a foraging zone. All information recorded was also the same as the social vs. foraging test, and to test for the development of an arm preference, both arms of the maze were empty during the fourth trial. Fifteen of the 31 chicks had the arm of the familiar social pair switched from the previous test to further discriminate potential arm bias. The same pairs of chicks were used as a stimulus and were not tested. In week 6, which coincided with trial 1 of the social recognition test, chicks in a ‘no hen’ pen were treated with an anticoccidial medication (Baycox, Bayer B.V. Kiel, Germany) following weight loss, and as prescribed by a veterinarian. The data from those animals were included for analysis as no behavioral abnormalities were noted after treatment.

#### 2.3.5. Holeboard

During weeks 9–11, all chicks were tested in the holeboard apparatus over 13 trials of 5 min, spread over 13 testing days (Figure 1C). The chicks were tested for 3 consecutive days, then for 6 consecutive days after a 2-day pause, and finally for 4 consecutive days after a 1-day pause. In every trial, all 9 cups were baited with 1 piece of grape; chicks had been exposed to grapes in the home cage, which they readily ate, at least 5 times prior to testing. Table 1 describes the measurements taken. Spatial working memory was calculated by dividing the number of grapes eaten in a trial by the total number of cup visits.

#### 2.3.6. Statistical Analysis

All statistics were calculated using SAS 9.4 English version software (SAS Institute Inc., Cary, NC, USA). Data were first tested for normal distributions. As the data from all behavioral tests were determined to be non-normally distributed, even following transformations, non-parametric testing was applied in the form of a Friedman Rank ANOVA. For one-trial behavioral tests (open field and voluntary human approach), scores for each variable were ranked and a two-way ANOVA (main effects rearing and genetic strain) was applied using PROC GLM. For the social versus foraging Y-maze and social recognition Y-maze, data from all trials were ranked for each variable. This procedure allows statistical testing of the effects of trials using rank data, which is not possible if ranks are computed per trial. Ranks were subsequently analyzed using a repeated-measures ANOVA using PROC GLM (between-subjects effects: rearing and genetic strain; within-subjects effect: trial). For holeboard data, first means per animal were computed for blocks of trials (Block 1: trials 1–5, Block 2: trials 6–9, and Block 3: trials 10–13). Computation of ranks and repeated measures ANOVA testing was then conducted as for the Y-mazes, using block means rather than trials.

Given the large number of variables tested, resulting in 330 *p*-values, a Benjamini–Hochberg procedure was performed based on a false discovery rate of 15%. This led to a q value of 0.009, corresponding to rank 21, and a p-value of 0.0085. A *p*-value of ≤ 0.0085 was thus considered significant.

## 3. Results

For all behavioral tests, relevant statistical results are reported below. See Supplemental Materials for a complete report of statistical results.

### 3.1. Open Field

**Figure 2:**
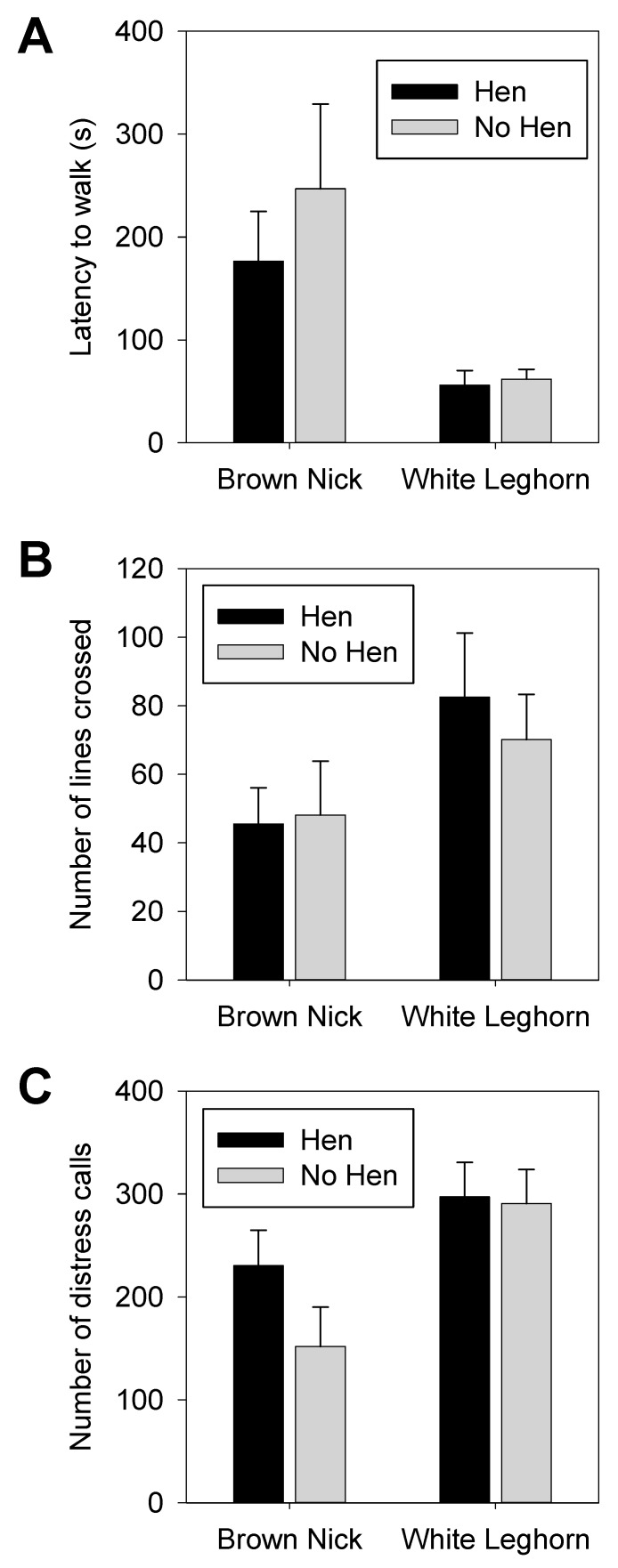
Results of open field testing for the parameters (**A**) latency to walk, (**B**) number of lines crossed, and (**C**) number of distress calls. Significant main effects of chicken line were found on latency to walk and number of distress calls. No significant effects of hen raising or line × hen raising interactions were observed. Bars represent averages and Standard Errror of the Mean (SEM).

No differences in behavioral reactions between chicks raised with a mother hen and those raised without a hen were found (latency to walk: F1,36 = 0.07, *p* = 0.79, number of lines crossed: F1,36 = 0.04, *p* = 0.83, number of distress calls: F1,36 = 0.93, *p* = 0.34, time to stop calling: F1,36 = 0.62, *p* = 0.44; Figure 2 and Table S1). However, the behavior of the two commercial strains differed significantly on two variables. The LH hens started walking earlier than the BN hens (latency to move: F1,36 = 17.49, *p* < 0.0005) and produced more distress calls during the test period compared to the BNs (F1,36 = 7.81, *p* <0.01. No statistically significant interactions were seen between rearing type and genetic strain on any of the variables tested in the open field.

### 3.2. Voluntary Human Approach Test

**Figure 3:**
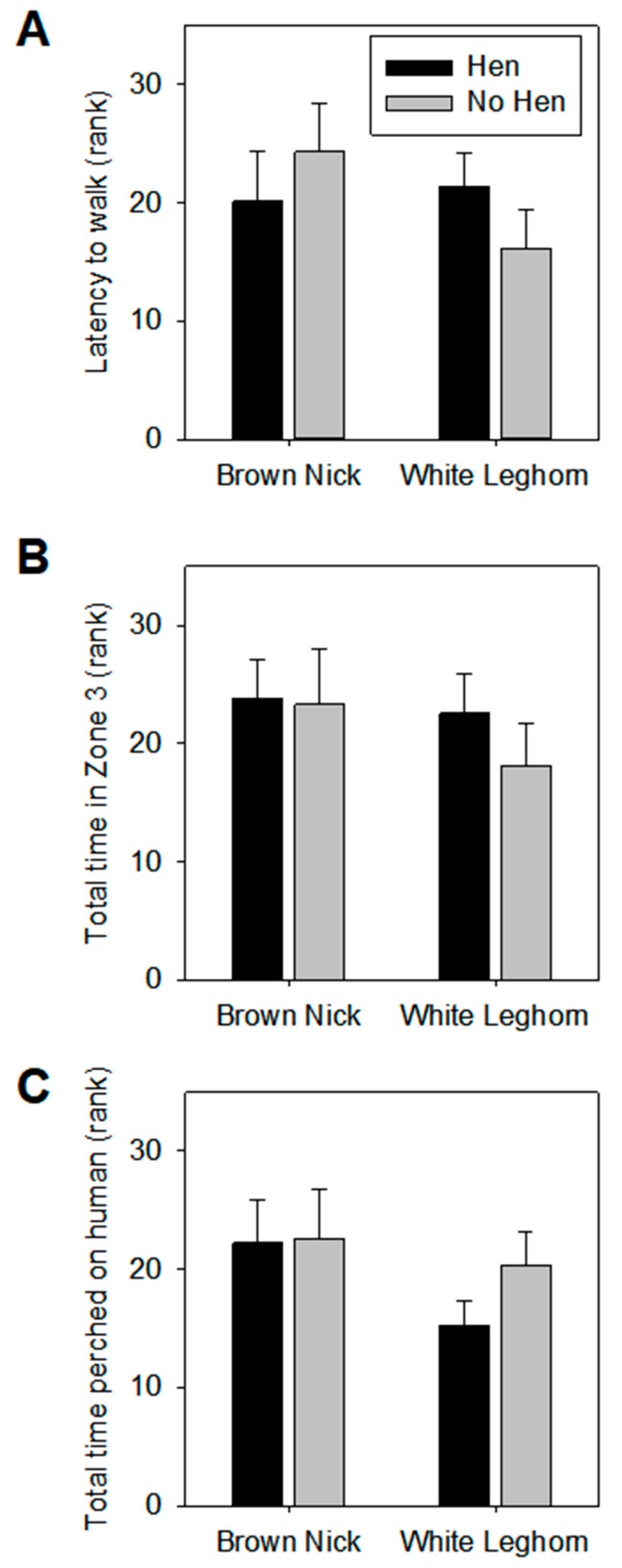
Results of the Voluntary Human Approach test for the parameters latency to walk, time in zone 3, and total time on human. No significant effects were seen on these parameters. Bars represent averages and SEM of ranked data.

Rearing with or without a hen did not affect the behavior of the chicks in the voluntary human approach test for any of the variables tested (see Figure 3 for representation of main measures and Table S2). Genetic strain affected only the re-entries into zone 1 (the starting area; F1,36 =10.10, *p* < 0.005). No other statistically significant effects of genetic strain or rearing–genetic interactions were found.

### 3.3. Social Versus Foraging Y-Maze

**Figure 4:**
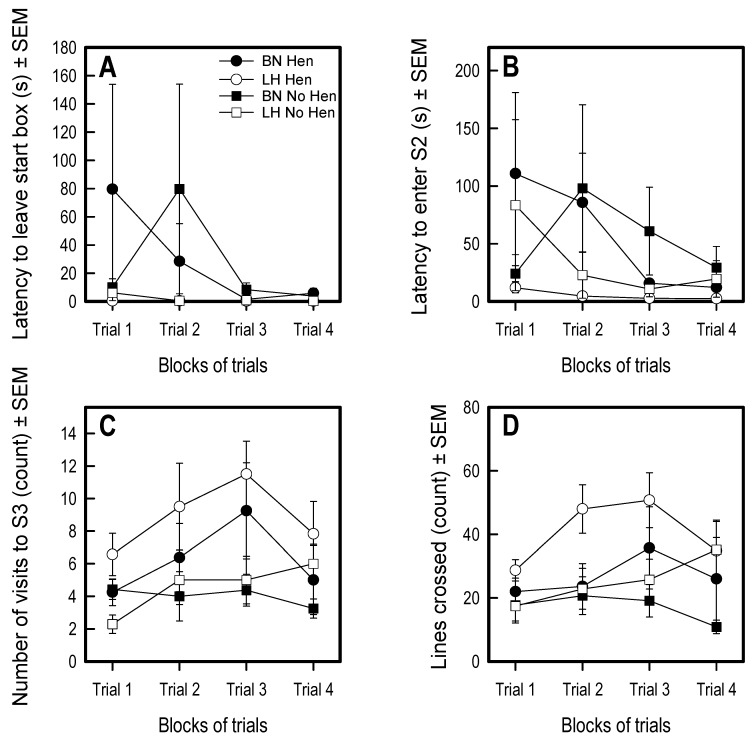
Results of the social vs. foraging test for the parameters (**A**) latency to leave start box, (**B**) latency to enter S2, (**C**) number of visits to S3, and (**D**) lines crossed. Significant main effects of line were found on latency to leave start box and latency to enter S2. Significant main effects of hen rearing were found on visits to S3 and lines crossed. Lines represent averages and SEM.

Overall, rearing condition had minimal effects on behavior in the social vs. foraging Y-maze (see Figure 4 for representation of main measures and Table S3). The only statistically significant main effect of rearing was found in visits to S3, the zone closest to conspecifics: hen-reared chicks re-entered the social zone more times than the chicks reared without a mother hen (F1,20 = 16.43, *p* < 0.001) (Figure 4C and Table S3).

On the other hand, genetic strain had significant effects on two latencies in the social vs. foraging Y-maze. The BN hens remained in the start box for longer (F1,20 = 30.95, *p* < 0001; Figure 4A and Table S3) and took longer to enter the areas with conspecifics compared to the LH hens (S2: F1,20 = 41.36, *p* < 0.001; S3: F1,20 = 9.03, *p* < 0.01; Figure 4B,C and Table S3). 

### 3.4. Social Recognition Y-Maze

**Figure 5:**
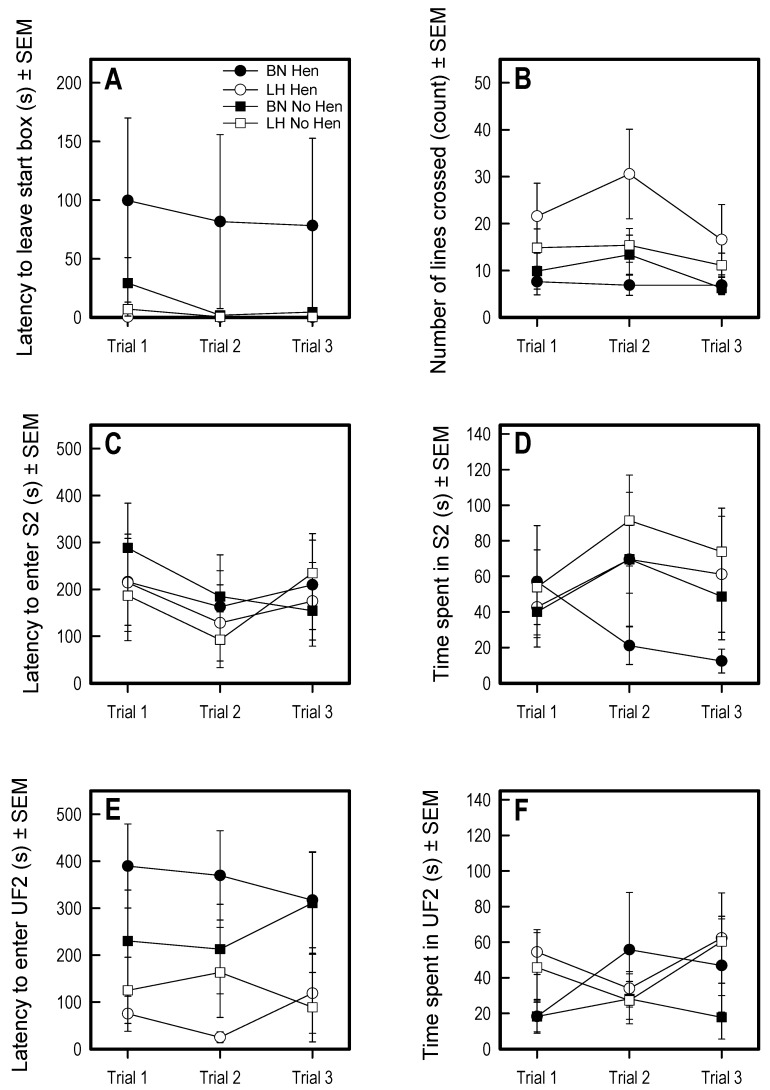
Relevant behavioral measures in the Social recognition Y-maze. Significant effects of genetic strain were seen in (**A**) latency to leave start box, (**B**) number of lines crossed, and (**E**) latency to enter UF2. No significant differences were seen in latency to enter (**C**) FA2 (**D**) total time in FA2, nor in (**F**) time spent in UF2; no significant effects of session or interactions were observed in any variable. Lines represent averages and SEM.

Rearing with or without a hen did not affect the behavior of the chicks in the social recognition test for any of the variables measured (see Figure 5 for representation of main measures and Table S4).

The LHs showed more activity, seen in more lines crossed than the BNs during the test (F1,27 = 9.66, *p* = 0.004). Consistent with the previous tests, the LHs were significantly faster out of the start box than the BNs (genetic: F1,27 = 31.34, *p* < 0.0001). The most prominent differences were seen in approach to the unfamiliar conspecifics, where the LHs showed shorter latencies than the BNs to approach the unfamiliar chickens (main effect line area UF2: F1,27 = 22.10, *p* < 0.0001; main effect line area UF3: F1,27 = 17.80; *p* = 0002). LHs visited the unfamiliar chickens (area UF3) more frequently than BN hens (F1,27 = 9.80, *p* = 0.004).

### 3.5. Holeboard Spatial Working Memory Task

**Figure 6:**
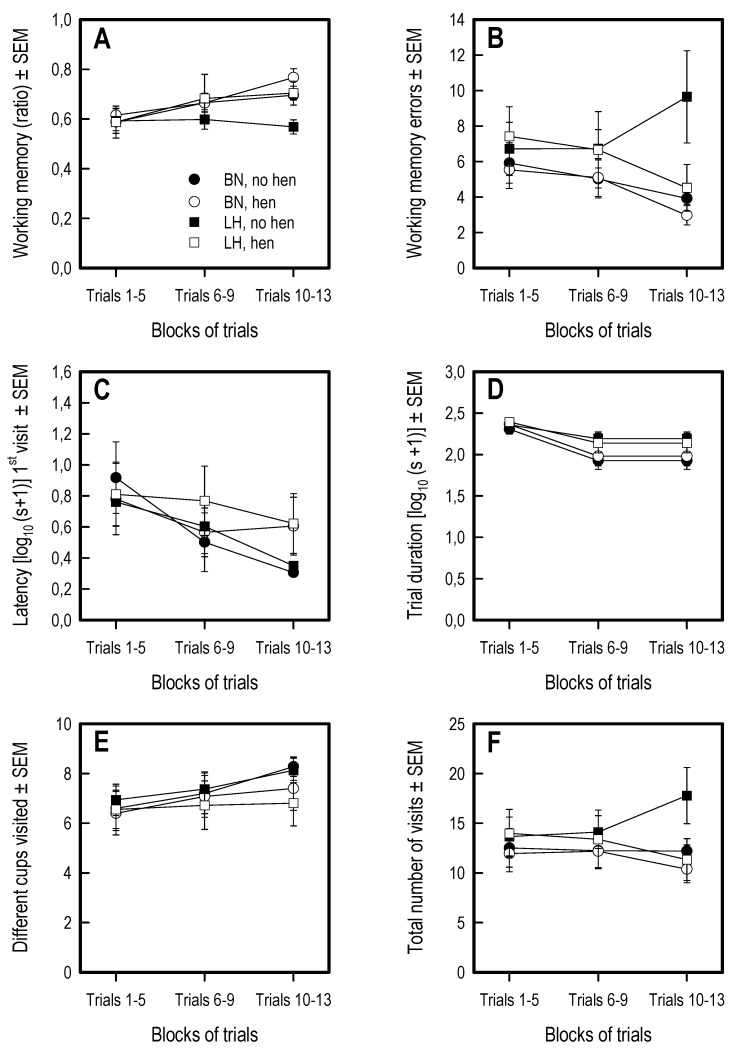
Relevant behavioral measures in the holeboard spatial working memory task. (**A**) Working memory ratio, (**B**) Working memory errors, (**C**) Latency to visit first cup (log values), (**D**) Trial duration (log values), (**E**) Different cups visited, (**F**) Total number of visits to cups (see Table 1 for complete explanation of variables). A trend to effect of genetic strain was found on working memory. A significant effect of session was found on working memory (ratio), latency to first visit, trial duration and different cups visited. No other main effects or interactions were observed. Lines represent averages and SEM.

Overall, all chicks visited the first cup quicker over trials (session: F 2,70 = 36.77, *p* < 0.001) (Figure 6C), decreased the trial time over sessions (session: F 2,70 = 31.53, *p* < 0.001) and visited more of the 9 cups over trial blocks (F 2,70 = 19.91, *p* < 0.001) (Figure 6E). See Figure 6 for representation of main measures and Table S5)

Neither genetic strain or hen rearing affected any of the variables tested, nor were there any interactions observed.

## 4. Discussion

Fear responses, motivation, and cognitive abilities were tested in two commercial hybrid lines of laying hen chicks. The effects of a foster mother hen on their behavior was also examined. Clear differences in the fear response to novelty and isolation from penmates, and social and exploratory motivation were found between the two commercial lines. In various novel environments, the LHs were consistently the first to move, were the most active during the testing periods, were faster to approach unfamiliar conspecifics, and spent more time foraging in an unfamiliar environment than the BN hens. The LHs also tended to perform better in the spatial memory holeboard task as well.

Rearing by a hen had little effect on many of the behavioral tests employed. The only significant effects were seen in the social vs. foraging Y-maze, where the hen-reared animals showed more activity and visited the area closest to conspecifics more than animals raised without a mother hen. Given the strong evidence that maternal care can have a profound effect on development of fear, social behaviors, and cognition, this general lack of effect in the present study was unexpected.

One limitation of the present study is the use of only female chicks. Keeping with practice on layer farms, we decided to only test layer hen chicks. Previous studies have shown that sex may play a role in sociality and cognition in chickens [50,51], and this should be kept in mind when generalizing the results of the present study. It should also be noted that maternal care was investigated only post-hatch; thus, the potential effects of maternal care and/or communication between the hen and chicks, were not included in the present study. Furthermore, we chose to use a breed of chicken that readily displays brooding and maternal care, given that commercial (hybrid) lines do not readily show brooding and/or maternal behaviors. As different breeds or hybrids of chicken clearly can display varying behaviors, this may have an effect on the type of maternal care that the chicks received. It would be of interest for future studies to determine whether the breed of foster mother has an effect on the behavior of the chicks.

### 4.1. Genetic Effects on Behavioral Reactions to Novel Situations

When the animals were 3 weeks old, they were tested in the classic open field fear test, and the two lines expressed different but equally adaptive fear responses for young, naive animals. The LHs were quick to search for an escape and to begin emitting distress calls, typifying a flight response. The BNs, on the other hand, took significantly longer to move and distress call, expressing an initial startled freezing response [21,52,53]. Despite familiarization across the apparatus in subsequent tests, and increases in age, the BNs maintained a more cautionary approach and were much slower to take a first step and moved much less throughout each experiment and trial compared to the LHs. Contrary to our hypothesis based on previous studies [18,19], by conventional analysis of animal behavior in response to novel situations, we would conclude that BNs are highly fearful, expressing a startled freezing response while LHs are less afraid and spend time exploring the new environment [21,52,53]. One could also hypothesize, based on the coping strategies described by Koolhaas et al. [54,55], that the BNs express a reactive or passive coping style by acting only when necessary, demonstrating a more adaptive approach with regards to predation risk avoidance [54,56]. However, the tendency for the LHs to perform better in the working memory holeboard task in combination with a more exploratory and bold response to novel contexts, demonstrates an equally adaptive approach to survival.

Our contradictory findings towards our prediction of LH fearful behavior based on a previous study further underlines the potential difficulties that can arise when classifying an animal as afraid or anxious based on their activity levels. Uitdehaag et al. [18] noted that low amounts of defecation, a signal of fear, did not correlate with the reduced activity and presumed higher levels of fear in the LHs. Nonetheless, Uitdehaag et al. [18] studied the open field behavior of young chicks of a similar age to the chicks in the present study but found that latency to walk was on average >500 s while in this study LHs started moving <65 s on average in each test and trial. The difference in behavior may be related to differences in housing conditions between the current study and the study by Uitdehaag et al., supporting further investigation of cage versus larger pen effects on specific laying hen strains.

### 4.2. Genetic Effects on Exploratory and Social Behavior

The LH hens were faster than BN hens to leave the start area in all tests, and LH hens showed shorter latencies to approach both foraging material and conspecifics in the social vs. foraging Y-maze compared to BN hens, fitting an active coping style to novelty [55,57]. Alternatively, LH foraging motivation could be quite strong, even during stressful situations, and should be considered in their housing. The LH hens readily sought social contact when in both novel and habituated environments and appeared more interested in the novel conspecifics than the BNs. The comfort of social contact for Leghorns is supported by Väisänen and Jensen [48], who also found young Leghorns prefer to be in close proximity with familiar conspecifics. More recently, social motivation was found to be greater in white egg layers compared to brown egg layers [12]. The BNs were consistently placid and seemingly unmotivated throughout the various tests, except when a familiar human was present. The clear difference in temperament and motivation of the two lines in this study further support the idea that the genetic background of hen to be used in a specific type of housing, should be taken into consideration to maximize laying hen welfare, as various genetic backgrounds may, i.e., have more or less motivation to join conspecifics or humans.

In the present study, no differences were seen between the two chicken lines in spatial working memory. In a previous (unpublished) study from our group, BN hens showed better working memory scores compared to Silver Nick hens [58]; thus, genetic background does seem to affect working memory performance in this test. In the present case, the differences may be too small, or the number of animals tested too small, to observe statistically significant differences.

### 4.3. Effects of Rearing on Sociality and Lack of Other Effects

In general, the effects of raising the chicks with a mother hen were unexpectedly absent in our behavioral tests. Contrary to predictions, cognitive performance was not altered by the presence of a mother hen in early life, rearing type did not reduce or alter fear responses, did not influence the chicks’ desire to explore unfamiliar conspecifics, nor did it increase the social or exploratory motivation of the BNs. This does correspond with previous results from our group [23] showing little effect of maternal care in Silver Nick layer hens on behavior in an open field test, voluntary human approach test, a Y-maze test for sociality, a food preference test, and a food rewarded Y-maze test, and also lack of effect in the open field by Perré et al. [9]. This lack of effect was attributed mainly to technical issues (too few hens per chick, mixing of beak trimmed and intact birds) in Angevaare et al. but given the current results, maternal care may simply not have a strong effect in layer hens on the behavioral tests conducted. As we lack formal data on the repertoire and amount of maternal care provided to each chick in this study, we cannot rule out that the overall results reflect variation in the early life experiences of the individual chicks within each group.

Commercial breeds of layer hens are perpetually in the laying phase of reproduction and never (or extremely rarely) enter the hormonal state of brooding, which normally leads to hormonal changes that produce the onset of maternal behavior [59]. The loss of maternal behavior that has accompanied selection on high egg production in commercial laying hen lines may potentially coincide with selection for the reduction of a need or response to maternal care of the chicks during postnatal development. Although neuroanatomical alterations in adult laying hens have been linked to experience of a mother hen as chicks [36,60]—a long-lasting effect seen in adult hens, as the hens only experienced a mother hen for the first 5 weeks of life—studies of the effect of genetics on the acceptance or the seeking of maternal care by the chicks themselves are lacking. There is a complex interaction between chicks and hen maternal behavior, seen in for instance the effects of brood size on maternal behavior in quail [61]. Future studies might examine whether chicks from lines selected for egg production have self-selected for viability in conditions without a (foster) mother hen.

## 5. Conclusions

The genetic background of the chickens in the present study had marked effects on tests of sociality and fear, while the lines showed minimal differences in cognition. Maternal care did not seem to affect the behaviors measured, which may indicate that the tests are not sensitive enough to detect differences, that maternal care was not sufficient, or that layer chicks are relatively insensitive to (lack of) maternal care.

## Figures and Tables

**Table 1:** The measurements recorded in the holeboard trials (Adapted from van der Staay et al. [49]).

Measure	Description
Number of different holes visited	Reflects exploratory motivation (and efficacy of exploring)
Number of revisits	Working memory errors
Spatial working memory	Number of rewarded visits/Total number of visits within a trial
Latency to first cup	Time into the circle of the first cup visited after release into the arena
	Reflects anxiety and foraging motivation
Trial duration	Time elapsed to find all baits or maximum trial duration (300 s)
Total visits	Reflect both exploratory motivation and poor memory

## Supplementary Materials

The following are available online at https://www.mdpi.com/2076-2615/9/7/454/s1, Table S1: Statistical results for open field test (Friedman ANOVA on ranked data), Table S2: Statistical results for voluntary human approach test (Friedman ANOVA on ranked data), Table S3A: Statistical results of between-subjects effects for social versus foraging Y-maze test (Friedman repeated measures ANOVA on ranked data), Table S3B: Statistical results of within-subjects effects for social versus foraging Y-maze test (Friedman repeated measures ANOVA on ranked data) Table S4A: Statistical results of between-subjects effects for social recognition Y-maze test (Friedman repeated measures ANOVA on ranked data), Table S4B: Statistical results of within-subjects effects for social recognition Y-maze test (Friedman repeated measures ANOVA on ranked data), Table S5A: Statistical results of between-subjects effects for holeboard test (Friedman repeated measures ANOVA on ranked data), Table S5B: Statistical results of within-subjects effects for holeboard test (Friedman repeated measures ANOVA on ranked data).
