# Supplementary material for: Effects of Maternal Care During Rearing in White Leghorn and Brown Nick Layer Hens on Cognition, Sociality and Fear
Source: Animals (Basel). 2019 Jul 18;9(7):454. doi: 10.3390/ani9070454 (PMC6680883; doi:10.3390/ani9070454)
Supplement: Supplementary file 1 [file animals-09-00454-s001.pdf]

Supplemental materials

For all statistical testing, significance is set to p≤0.085 following a False Discovery Rate statistical analysis (see Materials and Methods).

Table 1S. Statistical results for open field test (Friedman ANOVA on ranked data).

	Rearing type		Genetic strain		Rearing type x genetic strain	
**Variable**	***F***	**p**	***F***	**p**	***F***	**p**
Latency to walk	0.07	0.7884	**17.49**	**0.0002**	0.33	0.5705
Number of lines crossed	0.04	0.8348	4.81	0.0353	0.13	0.7160
Attempts to fly	0.45	0.5049	0.14	0.7153	0.36	0.5508
Number of calls	0.93	0.3414	**7.81**	**0.0085**	0.54	0.4681
Time to stop calling	0.62	0.4379	4.98	0.0323	0.01	0.9366

Note: For all variables and effects tested, df = 1, 36.

Table S2. Statistical results for voluntary human approach test (Friedman ANOVA on ranked data).

	Rearing type		Genetic strain		Rearing type x genetic strain	
**Variable**	***F***	**p**	***F***	**p**	***F***	**p**
Latency to walk	0.02	0.8929	0.85	0.3628	1.62	0.2108
Time to leave Zone 1	0.24	0.6279	0.14	0.7136	0.78	0.3819
Total time Zone 1	0.41	0.5285	0.46	0.5004	1.46	0.2356
Re-entries into Zone 1	0.82	0.3722	**10.10**	**0.0032**	0.01	0.9218
Time to enter Zone 3	0.81	0.3742	1.84	0.1834	0.46	0.5019
Total time Zone 3	0.01	0.9235	2.84	0.1008	0.06	0.8158
Total time perched on human	0.68	0.4164	2.09	0.1572	0.53	0.4698

Note: For all variables and effects tested, df = 1, 36.

Table 3. Statistical results of between-subjects effects for social versus foraging Y-maze test (Friedman repeated measures ANOVA on ranked data).

	Hen rearing		Genetic strain		Hen rearing x genetic strain	
**Variable**	***F***	**p**	***F***	**p**	***F***	**p**

latency (s) to leave start box	2.86	0.1061	**30.95**	**<.0001**	1.34	0.2605
total time (s) in Zone 1 (start arm)	2.13	0.1628	2.57	0.1275	0.02	0.9029
latency (s) to enter S2 (arm with conspecifics, distal to stimulus chickens)	0.29	0.5960	**41.36**	**<.0001**	0.22	0.6418
total time (s) in S2 (arm with conspecifics, distal to stimulus chickens)	0.00	0.9881	0.48	0.5000	0.01	0.9339
latency (s) to enter S3 (arm with conspecifics, proximate to stimulus chickens)	0.87	0.3620	**9.03**	**0.0070**	0.48	0.4985
total time(s) in S3 (arm with conspecifics, proximate to stimulus chickens)	0.10	0.7611	0.39	0.5387	0.09	0.7726
latency (s) to enter F2 (arm with foraging materials, distal foraging materials)	2.23	0.1514	4.09	0.0574	0.01	0.9055
total time (s) in F2 (arm with foraging materials, distal to foraging materials)	0.77	0.3924	0.12	0.7335	0.00	0.9832
latency (s) to enter F3 (arm with foraging materials, proximate to foraging materials)	1.38	0.2552	6.41	0.0203	0.05	0.8248
total time (s) in F3 (arm with foraging materials, proximate to foraging materials)	0.37	0.5491	7.66	0.0132	0.48	0.4989
latency (s) to enter foraging zone	1.59	0.2230	**9.95**	**0.0052**	1.93	0.1803
total time (s) in foraging zone	0.71	0.4111	0.71	0.4111	0.79	0.3859
number of lines crossed	**10.83**	**0.0043**	4.42	0.0508	0.38	0.5432
number of visits to S3	**16.43**	**0.0008**	3.04	0.0993	1.37	0.2588
Number of visits to foraging zone (FZ)	2.69	0.1193	7.93	0.0119	0.99	0.3326
total time (s) in arm with foraging material (F2 + F3 + foraging zone)	0.19	0.6668	3.38	0.0833	0.11	0.7499
total time (s) spent in midzone	0.22	0.6461	0.12	0.7328	0.20	0.6589
total time (s) in arm with conspecifics (S2 + S3)	0.19	0.6653	0.47	0.5013	0.16	0.6939

Note: For all between-subjects variables and effects tested, df = 1, 20.

Table S3b. Statistical results of within-subjects effects for social versus foraging Y-maze test (Friedman repeated measures ANOVA on ranked data).

	**Session**		**Session x Hen rearing**		**Session x Genetic strain**		**Session x Hen rearing x Genetic strain**	
**Variable**	***F***	**p**	***F***	**p**	***F***	**p**	***F***	**p**
latency (s) to leave start box	0.32	0.8083	0.41	0.7448	0.49	0.6910	1.58	0.2043
total time (s) in Zone 1 (start arm)	**4.74**	**0.0054**	1.46	0.2362	1.42	0.2489	0.12	0.9481
latency (s) to enter S2 (arm with conspecifics, distal to stimulus chickens)	2.05	0.1162	0.83	0.4829	0.16	0.9246	0.64	0.5898

total time (s) in S2 (arm with conspecifics, distal to stimulus chickens)	0.12	0.9462	0.97	0.4154	1.98	0.1290	0.03	0.9937
latency (s) to enter S3 (arm with conspecifics, proximate to stimulus chickens)	2.75	0.0507	1.22	0.3101	0.31	0.8199	0.64	0.5929
total time(s) in S3 (arm with conspecifics, proximate to stimulus chickens)	1.22	0.3108	1.76	0.1673	0.84	0.4776	0.39	0.7609
latency (s) to enter F2 (arm with foraging materials, distal foraging materials)	0.61	0.6094	0.29	0.8350	1.05	0.3785	0.53	0.6633
total time (s) in F2 (arm with foraging materials, distal to foraging materials)	0.21	0.8909	0.33	0.8024	0.10	0.9604	0.33	0.8070
latency (s) to enter F3 (arm with foraging materials, proximate to foraging materials)	0.88	0.4575	0.72	0.5426	1.06	0.3721	0.35	0.7911
total time (s) in F3 (arm with foraging materials, proximate to foraging materials)	0.12	0.9468	0.40	0.7508	0.13	0.9445	0.29	0.83
latency (s) to enter foraging zone	1.83	0.1521	0.80	0.4978	0.78	0.5083	0.19	0.9061
total time (s) in foraging zone	1.74	0.1703	0.13	0.9442	0.33	0.8017	0.34	0.8001
number of lines crossed	1.42	0.2484	0.50	0.6854	1.41	0.2518	0.06	0.9813
number of visits to S3	2.20	0.0989	0.31	0.8197	1.68	0.1834	0.69	0.5638
Number of visits to FZ	1.20	0.3189	0.96	0.4201	0.58	0.6327	0.23	0.8749
total time (s) in arm with foraging material (F2 + F3 + foraging zone)	0.64	0.5943	0.33	0.8036	0.39	0.7624	0.28	0.8417
total time (s) spent in midzone	1.00	0.3989	1.37	0.2618	0.35	0.7862	0.60	0.6186
total time (s) in arm with conspecifics (S2 + S3)	1.10	0.3590	1.56	0.2114	1.16	0.3339	0.52	0.6700

Note: For all within-subjects variables and effects tested, df = 3,60.

Table S4a. Statistical results of between-subjects effects for social recognition Y-maze test (Friedman repeated measures ANOVA on ranked data).

	**Hen rearing**		**Genetic strain**		**Hen rearing x genetic strain**	
**Variable**	*F*	p	*F*	p	*F*	p
latency to leave start box (s)	0.83	0.3692	31.34	<.0001	0.94	0.3402
total time (s) in Zone 1 (start arm)	0.00	0.9722	0.71	0.4069	0.29	0.5961
total time in start box + Z1 (s)	0.04	0.8503	0.16	0.6878	0.00	0.9511
latency (s)to enter area FA2 (arm with familiar chickens, distal to chickens)	0.03	0.8676	0.65	0.4277	0.01	0.9160
total time (s)in area FA2 (arm with familiar chickens, distal to chickens)	0.92	0.4065	3.01	0.0943	0.12	0.7290
latency (s)to enter area FA3 (arm with familiar chickens, proximate to chickens)	0.24	0.6295	0.07	0.7875	0.02	0.9028

total time(s)in area FA3 (arm with familiar chickens, proximate to chickens)	0.09	0.7631	0.11	0.7463	0.04	0.8337
latency (s)to enter area UF2 (arm with unfamiliar chickens, distal to chickens)	0.52	0.4751	**22.10**	**<.0001**	2.24	0.1462
total time (s) in area UF2 (arm with unfamiliar chickens, distal to chickens)	0.56	0.4625	7.96	0.0089	0.00	0.9927
latency (s)to enter area UF3 (arm w/ unfamiliar chickens, proximate to chickens)	0.43	0.5157	**17.80**	**0.0002**	3.12	0.0886
total time (s) in area UF3 (arm w/ unfamiliar chickens, proximate to chickens)	0.01	0.9179	2.47	0.1274	0.72	0.4039
number of lines crossed	0.01	0.9131	**9.66**	**0.0044**	1.09	0.3060
number of visits to FA3	0.03	0.8755	3.75	0.0633	0.52	0.4750
number of visits to UF3	0.91	0.3482	**9.80**	**0.0042**	0.83	0.3689
total time in arm with unfamiliar chickens (UF2 + UF3)	0.10	0.7512	2.74	0.1092	0.56	0.4619
total time spent in midzone	0.00	0.9633	3.71	0.0647	0.36	0.5554
total time in arm with familiar chickens (FA2 + FA3)	0.26	0.6121	0.08	0.7734	0.13	0.7208

Note: For all between-subjects variables and effects tested, df = 1, 27.

Table S4b. Statistical results of within-subjects effects for social recognition Y-maze test (Friedman repeated measures ANOVA on ranked data).

	**Block**		**Block x Hen rearing**		**Block x Genetic strain**		**Block x Hen rearing x genetic strain**	
**Variable**	***F***	**p**	***F***	**p**	***F***	**p**	***F***	**p**
latency to leave start box (s)	4.19	0.0203	0.89	0.4146	0.54	0.5832	0.14	0.8718
total time (s) in Zone 1 (start arm)	1.33	0.2722	0.70	0.5030	0.75	0.4760	2.43	0.0978
total time in start box + Z1 (s)	2.03	0.1416	0.46	0.6359	0.69	0.5075	0.99	0.3772
latency (s)to enter area S2 (arm with familiar chickens, distal to chickens)	2.53	0.0888	0.06	0.9401	1.34	0.2699	0.92	0.4065
total time (s)in area S2 (arm with familiar chickens, distal to chickens)	1.42	0.2498	0.90	0.4137	0.85	0.4340	0.33	0.7225
latency (s)to enter area S3 (arm with familiar chickens, proximate to chickens)	2.28	0.1116	0.23	0.7917	1.36	0.2646	0.64	0.5293
total time(s)in area S3 (arm with familiar chickens, proximate to chickens)	0.90	0.4140	0.22	0.8051	0.32	0.7265	1.41	0.2536
latency (s)to enter area UF2 (arm with unfamiliar chickens, distal to chickens)	0.40	0.6705	0.01	0.9905	1.34	0.2703	1.02	0.3657
total time (s) in area UF2 (arm with unfamiliar chickens, distal to chickens)	0.14	0.8717	0.02	0.9794	1.40	0.2543	0.69	0.5039

latency (s)to enter area UF3 (arm w/ unfamiliar chickens, proximate to chickens)	0.05	0.9479	0.00	0.9998	0.43	0.6533	0.81	0.4514
total time (s) in area UF3 (arm w/ unfamiliar chickens, proximate to chickens)	0.49	0.6124	0.13	0.8819	0.85	0.4331	1.59	0.2142
number of lines crossed	1.98	0.1484	0.22	0.8047	0.10	0.9043	0.72	0.4898
number of visits to S3	3.83	0.0279	0.24	0.7853	0.50	0.6111	0.39	0.6758
number of visits to UF3	0.20	0.8212	0.25	0.7811	0.26	0.7715	1.72	0.1892
total time in arm with unfamiliar chickens (UF2 + UF3)	0.56	0.5760	0.03	0.9750	0.86	0.4296	1.39	0.2571
total time spent in midzone	0.27	0.7611	0.25	0.7765	1.89	0.1616	0.50	0.6106
total time in arm with familiar chickens (S2 + S3)	0.95	0.3927	0.15	0.8622	0.34	0.7103	1.61	0.2085

Note: For all within-subjects variables and effects tested, df = 2,54.

Table S5a. Statistical results of between-subjects effects for holeboard test (Friedman repeated measures ANOVA on ranked data).

	Hen rearing		Genetic strain		Hen rearing x genetic strain	
**Variable**	***F***	**p**	***F***	**p**	***F***	**p**
Latency to first cup (s)	1.20	0.2811	1.06	0.3102	0.00	0.9721
Total trial time (s)	0.48	0.4916	1.83	0.1853	0.00	0.9727
Number of different cups visited	0.45	0.5086	0.42	0.5230	0.06	0.8091
Total number of visits to cups	0.64	0.4289	2.53	0.1206	0.14	0.7128
Total number of revisits to cups	0.67	0.4193	2.76	0.1054	0.22	0.6449
Number of grapes eaten	0.62	0.4375	0.97	0.3313	0.08	0.7808
Working memory	1.06	0.3100	3.76	0.0608	0.07	0.7934

Note: For all between-subjects variables and effects tested, df = 1,35.

Table S5b. Statistical results of within-subjects effects for holeboard test (Friedman repeated measures ANOVA on ranked data).

	Block		Block x Hen rearing		Block x Genetic strain		Block x Hen rearing x genetic strain	
**Variable**	***F***	**p**	***F***	**p**	***F***	**p**	***F***	**p**
Latency to first cup (s)	**36.77**	**<.0001**	**5.32**	**0.0071**	1.13	0.3277	0.42	0.6561
Total trial time (s)	**31.53**	**<.0001**	0.01	0.9924	2.29	0.1088	0.37	0.6895
Number of different cups visited	**19.91**	**<.0001**	0.28	0.7578	1.06	0.3518	0.10	0.9023
Total number of visits to cups	2.18	0.1206	2.03	0.1385	1.65	0.1986	1.69	0.1918
Total number of revisits to cups	4.72	0.0119	2.96	0.0585	2.22	0.1167	1.25	0.2935
Number of grapes eaten	17.16	<.0001	0.38	0.6824	0.80	0.4525	0.14	0.8729
Working memory	9.02	0.0003	1.90	0.1573	2.13	0.1264	1.35	0.2673

Note: For all within-subjects variables and effects tested, df = 2,70.
